# A novel antigen biomarker for detection of high-level of *Loa loa* microfilaremia

**DOI:** 10.1371/journal.pntd.0012461

**Published:** 2024-09-03

**Authors:** Sarah E. Greene, Yuefang Huang, Kerstin Fischer, Bruce A. Rosa, John Martin, Makedonka Mitreva, Devyn Yates, Samuel Wanji, Joseph Kamgno, Philip J. Budge, Gary J. Weil, Peter U. Fischer

**Affiliations:** 1 Infectious Diseases Division, Department of Pediatrics, Washington University School of Medicine, St Louis, Missouri, United States of America; 2 Infectious Diseases Division, Department of Medicine, Washington University School of Medicine, St Louis, Missouri, United States of America; 3 Department of Genetics, Washington University School of Medicine, St Louis, Missouri, United States of America; 4 McDonnell Genome Institute, Washington University School of Medicine, St Louis, Missouri, United States of America; 5 Parasites and Vector Biology research unit (PAVBRU), Department of Microbiology and Parasitology, University of Buea, Buea, Cameroon; 6 Research Foundation for Tropical Diseases and the Environment (REFOTDE), Buea, Cameroon; 7 Higher Institute for Scientific and Medical Research (ISM), Yaoundé, Cameroon; 8 Department of Public Health, Faculty of Medicine and Biomedical Sciences, Department of Public Health, University of Yaoundé I, Yaoundé, Cameroon; George Washington University School of Medicine and Health Sciences, UNITED STATES OF AMERICA

## Abstract

**Background:**

Loiasis is a disease caused by the nematode *Loa loa*. Serious adverse events sometimes occur in people with heavy *L*. *loa* microfilaremia after ivermectin treatment. In regions of Central Africa where loiasis is endemic, this significantly impedes global elimination programs for lymphatic filariasis and onchocerciasis that use mass distribution of ivermectin. Improved diagnostic tests to identify individuals at increased risk of serious adverse events could facilitate efforts to eliminate lymphatic filariasis and onchocerciasis in this region.

**Methods and findings:**

We previously identified the *L*. *loa* protein Ll-Bhp-1 in loiasis patient sera. Here, we further characterize Ll-Bhp-1 and report development of an antigen capture ELISA to detect this antigen. This assay detected Ll-Bhp-1 in 74 of 116 (63.8%) loiasis patient sera. Ll-Bhp-1 levels were significantly correlated with *L*. *loa* microfilarial counts, and the sensitivity of the assay was highest for samples from people with high counts, (94% and 100% in people with ≥20,000 and ≥50,000 microfilaria per milliliter of blood, respectively). The antigen was not detected in 112 sera from people with other filarial infections, or in 34 control sera from the USA.

**Conclusions:**

This Ll-Bhp-1 antigen assay is specific for loiasis, and highly sensitive for identifying people with high *L*. *loa* microfilarial counts who are at increased risk for serious adverse events after ivermectin treatment. *L*. *loa* antigen detection has the potential to facilitate loiasis mapping efforts and programs to eliminate lymphatic filariasis and onchocerciasis in Central Africa.

## Author summary

Lymphatic filariasis and onchocerciasis are major neglected tropical diseases that have been targeted for elimination by the World Health Organization. Elimination campaigns for these infections using mass drug administration have had a huge impact in Africa. However, they face a major challenge in 11 countries in Central Africa where there is co-endemic loiasis, another filarial infection caused by the parasite *Loa loa*. The anti-filarial medication ivermectin that is used in elimination programs can cause serious and fatal adverse drug events in people with heavy loiasis infections, or high blood microfilarial counts. These programs would benefit from practical and affordable screening tests to detect people at high risk of adverse events from ivermectin. Furthermore, loiasis itself causes a variety of negative health outcomes and improved diagnostic tests would help control this infection. Here, we report the development of a sandwich immunoassay that detects the *L*. *loa* protein Ll-Bhp-1 in human serum samples. This assay is specific for *L*. *loa* infection and is highly sensitive in people with high microfilarial counts. Further work is needed to refine this prototype test and to determine whether antigen testing for loiasis can facilitate filarial eliminations efforts in Central Africa.

## Introduction

Loiasis is an infection caused by the filarial nematode *Loa loa*. Loiasis is transmitted by *Chrysops* deer flies. Flies ingest microfilariae (Mf), the small larval stage of the parasite, when they feed on the blood of an infected person. Mf develop in the fly into infective third stage larvae that can be transmitted to humans. Loiasis can present with transient angioedema or eyeworm, wherein adult worms migrate under the bulbar conjunctiva. Loiasis sometimes causes marked hyper-eosinophilia and is correlated with endocardial fibrosis, poor cardiovascular outcomes, and increased all-cause mortality [1–4]. While *L*. *loa* infection can be asymptomatic, there is increasing awareness of the breadth of clinical problems that this infection can produce besides eyeworm and angioedema, such as arthralgias, myalgias, paresthesia, fatigue, and severe headaches [5]. One study reported an association between heavy *L*. *loa* microfilarial burden and altered cognition [6].

In addition to its effects on individuals’ health, *L*. *loa* infection also constrains public health programs that aim to eliminate the neglected tropical diseases lymphatic filariasis (LF) and onchocerciasis. These elimination programs are based on mass drug administration (MDA) of ivermectin, either alone or combined with albendazole. Serious neurologic complications including coma and death can occur when people with very high *L*. *loa* Mf counts are treated with ivermectin or other microfilaricidal drugs [7,8]. Ivermectin is associated with migration of Mf into the cerebrospinal fluid [9]. There may also be blockage of capillaries by dying Mf resulting in retinal hemorrhage, and an inflammatory response to dying Mf that causes neurologic complications [10–12]. Therefore, a “test-and-not-treat” approach has been proposed, wherein people in areas co-endemic for loiasis and LF or onchocerciasis are tested for daytime *L*. *loa* Mf burden and excluded from MDA if they have high *L*. *loa* Mf levels. The exact Mf cutoff for safe treatment with ivermectin in loiasis patients is unknown. The relative risk for clinically significant adverse events is increased in people who have >8,000 *L*. *loa* Mf/ml, however the highest risk is in individuals with >50,000 Mf/ml [7]. Other authors have proposed lower Mf cutoffs for serious post-treatment neurological adverse reactions [12]. Accurate and practical methods for detecting individuals with high *L*. *loa* Mf levels are needed to ensure these high-risk individuals are not given MDA.

Existing antibody-based diagnostics have varying sensitivity and specificity for diagnosis of loiasis [13,14]. However, antibody tests do not identify people with heavy infection burden who are at risk for serious adverse events. In fact, antibody levels can be inversely proportional to Mf count: people with high Mf burden often have lower antibody levels than people with lower Mf counts or occult amicrofilaremic loiasis [15]. Several *L*. *loa* antigens have been proposed as potential biomarkers of infection, and prototype tests have reported variable sensitivity and specificity, but no diagnostic tests are commercially available [16,17]. The LoaScope is a portable microscopy-based technique to identify people with high *L*. *loa* Mf counts [18]. The LoaScope has been used in a large-scale clinical trial to evaluate the test-and-not-treat strategy for onchocerciasis [19]. In a LoaScope test-and-not-treat trial, 15,000 people were screened and no adverse events were reported when a Mf cutoff for treatment of 20,000 Mf/ml was used, and hundreds of people with Mf counts between 8,000 and 20,000 were treated [19].

This study was prompted by the need for more accurate point of care diagnostics for heavy loiasis infection that might be practical for widespread use in low resource settings. A proteomic analysis identified parasite proteins in extracellular vesicles isolated from the serum of people with high *L*. *loa* Mf counts. Several potential biomarkers were identified, including one protein, encoded by gene EN70_10600, that was found in extracellular vesicles from all 10 loiasis serum samples tested [20]. Furthermore, a closely related paralogue, encoded by gene EN70_10598, was detected in vesicles from 6 of 10 serum samples tested [20]. These two antigens are different from previously reported *L*. *loa* biomarkers [16,17]. Both proteins are homologues of BmR1, an Mf-associated diagnostic antigen from the filarial parasite *Brugia malayi*. We have previously identified EN70_10598 and named the protein it encodes Ll-Bhp-1, for *L*. *l**oa*-BmR1 homologous protein-1 [21]. Therefore, the purposes of the present study were to further characterize Ll-Bhp-1 and to develop an immunoassay to assess the potential value of this antigen as a biomarker for *L*. *loa* infections, with a special focus on identification of high-Mf density infections that increase the risk of serious adverse events after ivermectin treatment.

## Methods

### Ethics statement

We used legacy sera samples collected as outlined in the studies listed in Table 1. Loiasis sera were collected after approval from the Cameroon National Ethics Committee and Ministry of Public Health, and by the institutional review board of Washington University in St Louis (protocol numbers 201512112, 201512016, and 201909003). All other sera were de-identified and the metadata regarding infection status and treatment history were only linked by study identification number. The Washington University in St Louis Human Research Protection Office (an institutional review board) determined that work with such de-identified samples did not constitute human subjects research.

**Table 1:** Sera characteristics.

Infection	Location of sera collection	Number of sera	Filarial and non-filarial co-infections	Diagnosis method	Ref
*Loa loa*	Cameroon	116	19 with *Mansonella perstans* (26%) 1 with *Trichuris trichiura* 1 with *Ascaris lumbricoides*	Daytime Mf counts by 50ul thick blood smear	[30,31]
*Onchocerca volvulus*	Uganda	47	*M*. *perstans* possible *Mansonella streptocerca* possible	Mf counts of skin snips	[32]
*Wuchereria bancrofti*	Sri Lanka	11	Absent	Nighttime Mf counts of 250 ul of filtered blood	[33]
	Egypt	7	Absent	Nighttime Mf count of 1ml of venous blood by membrane filtration	[34]
	India	7	Absent	Nighttime Mf count of 20 ul thick blood smear or 1ml of blood by membrane filtration	[35]
	C*ô*te d’Ivoire	11	Absent	Nighttime Mf count of 1ml of blood by membrane filtration	[36]
*Mansonella perstans*	Uganda	29	Absent	Daytime Mf count of blood smear of 100ul of buffy coat and modified Knott method	[37]
Non-endemic control	St. Louis, USA	34	Absent ^a^	Not applicable	[21]

^a^ Non-endemic control sera from the USA are presumed to be free from filarial infections.

### BmR1 homologue characterization

We used Clustal W in MegAlign version 15 (DNAStar, Madison WI, USA) to compare amino acid sequences for the *L*. *loa*, *W*. *bancrofti* and *O*. *volvulus* homologues of *B*. *malayi* BmR1. The bootstrap consensus tree was constructed in MEGA11 [22]. It was inferred from 1,000 replicates using the Maximum Likelihood method.

### Analysis of gene duplication in the *L*. *loa* genome

The current *L*. *loa* genome assembly and annotation (PRJNA246086) [23] was downloaded from WormBase Parasite (WBPS15) [24]. BLAST [25] (version 2.13.0+) was used to identify the percentage of amino acid sequence similarity between proteins of interest from the proteomics results. Long PacBio genomic sequence reads were retrieved from the previously published *Loa loa* genome paper [23] and were mapped to the current annotation of *Loa loa* using Minimap2 (v2.26) [26]. Genomic read coverage over regions of interest from two scaffolds (scf7180000007487_1 and scf7180000007489_1) was visualized using the Integrative Genomics Viewer (IGV, version 2.16.2) [27,28], providing depths of total coverage and the identification of reads spanning multiple genes of interest. IGV was also used to visualize gene and exon positions, as well as repeat families and low complexity regions along the length of the regions of interest. All genomic features were annotated using the generic feature format (GFF) files available for the current genome version (PRJNA246086) [23]. Long stretches of sequence similarity were identified along regions of interest using genome alignment program Mauve [29].

### Human samples

We used de-identified legacy serum or plasma samples from individuals with the listed filarial infections unless otherwise noted. We tested samples from people with *L*. *loa*, *W*. *bancrofti*, or *O*. *volvulus* infections (Table 1) We also tested de-identified sera from non-endemic controls that were obtained from the Barnes Jewish Hospital clinical laboratory in St. Louis, MO [21]. These samples were presumed to be from people free of filarial infections given the very low rates of travel to endemic areas and of diagnosed filarial infections in this region, although no clinical data were available for these samples.

### Cloning and protein production

The protein product of gene EN70-10598 was previously named Ll-Bhp-1. EN70-10598 was cloned into the expression vector pET100D, and Ll-Bhp-1 was expressed and purified as previously described [21].

### Antibody production

BALB/c mice were immunized with ~10 ug of recombinant Ll-Bhp-1 in complete Freund’s adjuvant then boosted with 20ug of recombinant Ll-Bhp-1 in incomplete Freund’s adjuvant 2 and 4 weeks after primary immunization. Mouse sera were collected 3 weeks after the second boost. This work was done according to protocols approved by the Washington University in St Louis Institutional Animal Care and Use committee (#A-3381-01). Polyclonal rabbit antibodies to Ll-Bhp-1 were commercially produced using recombinant Ll-Bhp-1 and purified by protein A affinity chromatography (LifeTein, Somerset, NJ, USA).

### Parasite Fixation and Immunohistochemistry

Adult *L*. *loa* worms were produced at the Research Foundation in Tropical Diseases and Environment in Buea, Cameroon in immunodeficient, lymphopenic mice as previously described [38]. Adult worms were fixed in 80% ethanol and embedded in paraffin. For immunohistochemistry, consecutive sections were stained using the alkaline phosphatase anti-alkaline phosphatase (APAAP) method as previously described [21,39]. Rabbit polyclonal antisera raised against recombinant Ll-Bhp-1 was used as the primary antibody at a dilution of 1:5,000. Pre-immunization sera from the same rabbit was used as the negative control, also at a dilution of 1:5,000.

### Antigen-capture ELISA to detect Ll-Bhp-1 in human sera

We incubated 200 μg of rabbit polyclonal anti-Ll-Bhp-1 in 1ml of citrate buffer pH 2.6 for 10 minutes at room temperature. This was then diluted into 9 ml in 0.1M carbonate buffer pH 8 to produce 20 μg/ml of antibody and used to coat 96 well ImmunoGrade high binding polystyrene round bottom plates (Brand, Wertheim, Germany), which were incubated overnight at 37°C. Plates were then washed 5 times in phosphate buffered saline with 0.05% Tween 80 (PBST) and blocked with PBST with 5% heat inactivated fetal calf sera (ELISA diluent) for 2 hours at 37°C. Plates were then coated with either serial dilutions of recombinant Ll-Bhp-1 as positive controls, ELISA diluent as a no antigen negative control, or patient sera. Before use, 23 μl of patient sera was mixed with 103 μl of ELISA diluent and 103 μl of 0.1M EDTA, heated to 100°C for 5 min then centrifuged for 5 min. 100 μl of this 1:10 dilution of sera was used for each well and all samples were tested in duplicate. Plates were incubated overnight at 37°C. Plates were washed 5 times in PBST then incubated with mouse anti-Ll-Bhp-1 at a concentration of 1:1000 in ELISA diluent at 37°C for 2 hours. Plates were again washed 5 times in PBST and then incubated for 2 hours at 37°C with horseradish peroxidase-conjugated anti-mouse IgG (Southern Biotech, Birmingham, Alabama, USA) diluted to 1:4000 in ELISA diluent. Plates were again washed 5 times in PBST then incubated in 100 μl of o-phenylenediamine dihydrochloride for 10 min. The enzymatic reaction was stopped with 50 μl of 4M H_2_SO_4_ and plates were read at 490 nm with a BioTek ELx808 plate reader (Thermo Fisher Scientific, Waltham MA, USA) to obtain optical density (OD) values. Mean OD values of the negative control wells without antigen or sera were used to blank the plates. Indicated datapoints represent the mean of duplicate well OD values.

### Statistical analysis

Statistical analyses were conducted with Excel v16.80 (Microsoft, Redmond, WA, USA) and Prism Version 9 software (GraphPad, Boston, MA, USA).

## Results

### Analysis of genes that encode *L*. *loa* Bmr1 homologues and related proteins

**Fig 1:**
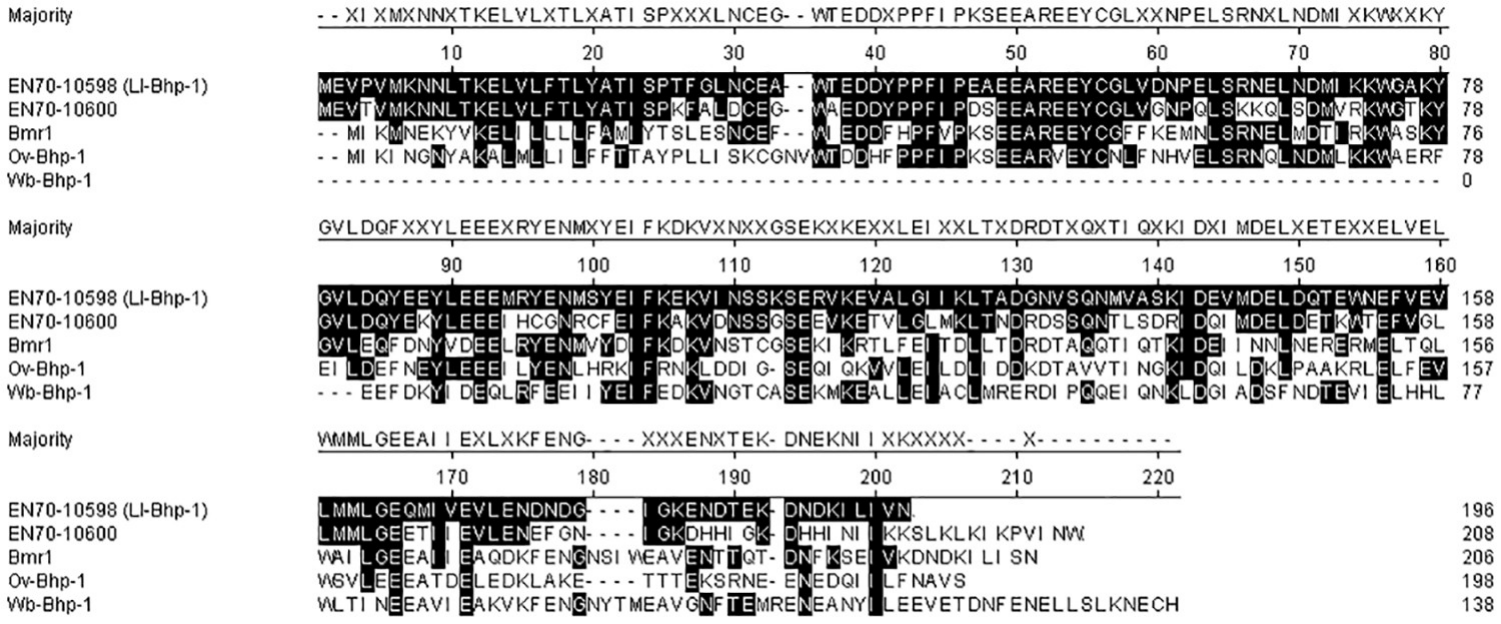
Alignment of BmR1 homologues. Amino acid alignment by Clustal W in MegAlign of EN70-10598, also known as Ll-Bhp-1, from *L*. *loa*, EN70-10600 from *L*. *loa*, BmR1 from *B*. *malayi*, Ov-Bhp-1, the BmR1 homologue from *O*. *volvulus*, and Wb-Bhp-1, the BmR1 homologue from *W*. *bancrofti*. Amino acid residues conserved with Ll-Bhp-1 are shaded black.

**Fig 2:**
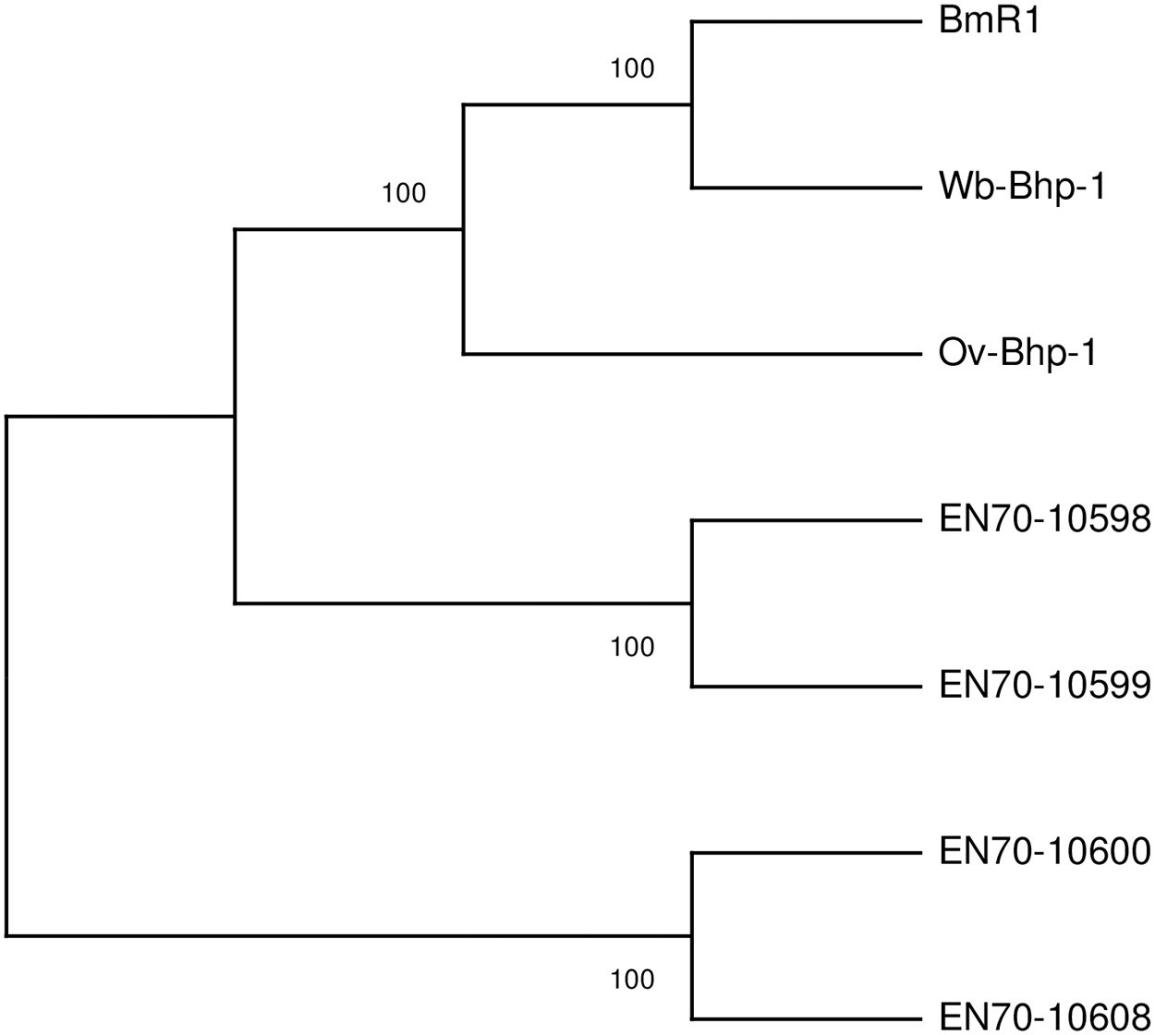
Phylogenetic tree of BmR1 homologues. The figure demonstrates the Maximum Likelihood phylogenetic tree of genes encoding BmR1 homologues from *B*. *malayi*, *O*. *volvulus*, *W*. *bancrofti* and *L*. *loa*. The tree was inferred from 1000 replicates. The number at each branchpoint indicates the percent of replicate trees where the named genes clustered together.

We previously identified two related *L*. *loa* proteins, encoded by genes EN70-10600 and EN70-10598, in extracellular vesicles isolated from patient sera [20]. These genes are homologues of the gene encoding the diagnostic antigen BmR1 in *B*. *malayi*. We further investigated the potential of these proteins as loiasis biomarkers. Three of the genes corresponding to these proteins (EN70_10598, EN70_10599 and EN70_10600) are positioned together on the same genomic scaffold (scf7180000007487_1, "Scaffold 7487"), with EN70_10598 and EN70_10599 sharing 100% sequence conservation. Another similar gene (EN70_10608) on scaffold scf7180000007489_1 ("Scaffold 7489") shared 99% of its sequence with EN70_10600. EN70_10600 and EN70_10608 shared 67% sequence similarity with EN70_10598 and EN70_10599 (S1A Fig). In order to verify whether these represent true gene duplication events as opposed to assembly errors, long PacBio genomic *L*. *loa* reads from the previously published *L*. *loa* genome [23] were mapped to the genome and were visualized. Mapped read depths were consistent across the length of the genomic regions of interest, and many reads spanned across all three genes on Scaffold 7487 (S2 Fig), indicating that these do indeed represent true gene duplication events. Read coverage on Scaffold 7489 also showed no indication that the gene was a miss-assembly, with consistent depth and reads spanning both upstream and downstream regions of EN70_10608 (S2 Fig). Genomic alignments indicated conservation of the gene sequences, as well as upstream regions containing repeats and regions of low complexity (S1B Fig). This finding suggests wider genomic duplication events. Taken together, these results provide confidence that these similar gene models represent distinct genes and proteins rather than assembly errors. In addition to these homologues in *L*. *loa*, BmR1 also has homologues in *O*. *volvulus* and *W*. *bancrofti* that we have previously described and named Ov-Bhp-1 and Wb-Bhp-1, respectively [21]. The alignment of these proteins is shown in Fig 1. As predicted, the paralogues encoded by EN70-10598 (Ll-Bhp-1) and EN70-10600 are more similar to each other than the BmR1 homologues from other filarial species. Similarly, a phylogenetic analysis of these proteins demonstrates that the *B*. *malayi* BmR1 is closer to its homologues from *W*. *bancrofti* and *O*. *volvulus* than to the homologues in *L*. *loa* (Fig 2).

### Immunohistochemical localization of Ll-Bhp-1

**Fig 3:**
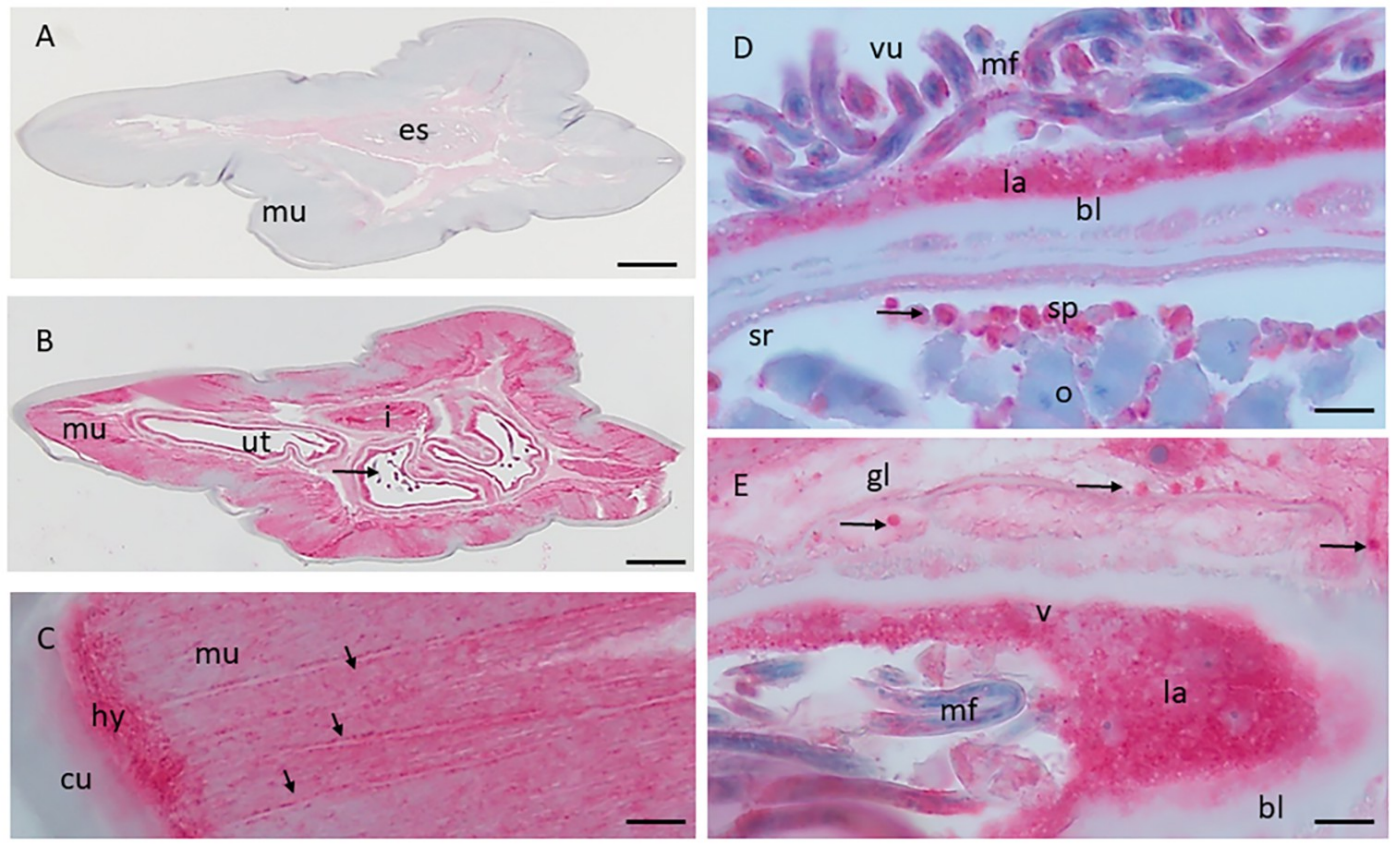
Immunolocalization of Ll-Bhp-1 in *L*. *loa* female worm. **A:** Anterior transverse section stained with pre-immune sera. **B:** Anterior transverse section, consecutive section to that in **A**, including anterior and posterior uterus, stained with post-immunize sera. Arrow indicates stretched microfilariae. **C:** Transverse section of the muscle. Arrows indicate the septa. **D:** Transverse section of the uterus. Arrow indicates spermatids. **E:** Transverse section of the vagina vera. Arrows indicate secretory granules with stored glycogen. Scale bars A-B 100 μm, C-E 10 μm. Abbreviations: **bl**, basal lamina; **cu**, cuticle; **la**, basal labyrinth; **gl**, glycogen; **mf**, microfilariae; **mu**, muscle; **es**, esophagus; **i**, intestine; **ut**, uterus; **hy**, hypodermis; **vu**, vagina uterine; **v**, vagina vera; **o**, oocytes; **sp**, spermatids.

To better characterize Ll-Bhp-1, we localized the antigen in adult *L*. *loa* worms using immunohistochemistry. *L*. *loa* anatomy has been previously characterized [40]. Immunolocalization showed strong labeling in many tissues of the adult worms and in Mf (Fig 3). No staining in the body wall or the digestive tract was observed in the negative control with pre-immune sera (Fig 3A). However, post-immune sera demonstrated strong labeling of the hypodermis and body wall muscles, especially along the muscle septa (Fig 3B and 3C). The basal labyrinth of the uterus is labeled, while the basal lamina of the seminal receptacle shows no labeling (Fig 3D). Anti-Ll-Bhp-1 antibody also bound to the spermatids and intrauterine Mf (Fig 3D and 3E), and the basal labyrinth of the vagina vera with strong labeling of secretory granules in that organ (Fig 3E).

### Detection of Ll-Bhp-1 in sera from individuals with loiasis

To assess the potential of Ll-Bhp-1 as a loiasis biomarker in blood, we developed an antigen-capture ELISA that could detect recombinant Ll-Bhp-1 (rLl-Bhp-1). A positivity cutoff of OD_490_ ≥ 0.2 was selected to optimize sensitivity and specificity, based on results of a receiver-operating characteristic (ROC) analysis (S3 Fig). The area under the ROC curve is 0.9748 with p<0.0001. This assay had a limit of detection of ~1 ng/ml for rLl-Bhp-1 (S4A Fig). We demonstrated that rLl-Bhp-1 is a heat stable antigen (S4 Fig). It has been previously shown that, for heat stable proteins, heating samples in EDTA can improve results for antigen assays [41]. Therefore, we tested sera with and without EDTA/ heat treatment and found this treatment tended to increase the ELISA OD values for loiasis sera without affecting OD values from control sera (S4 Fig). Treating with heat and low pH-glycine has also been shown to release antigen from immune complexes and has been previously used in other loiasis antigen assays [17]. However, that method did not increase assay sensitivity for Ll-Bhp-1 beyond what was seen with EDTA/ heat treatment (S4B Fig). Therefore, we utilized the EDTA/ heat method for our antigen assay.

**Fig 4:**
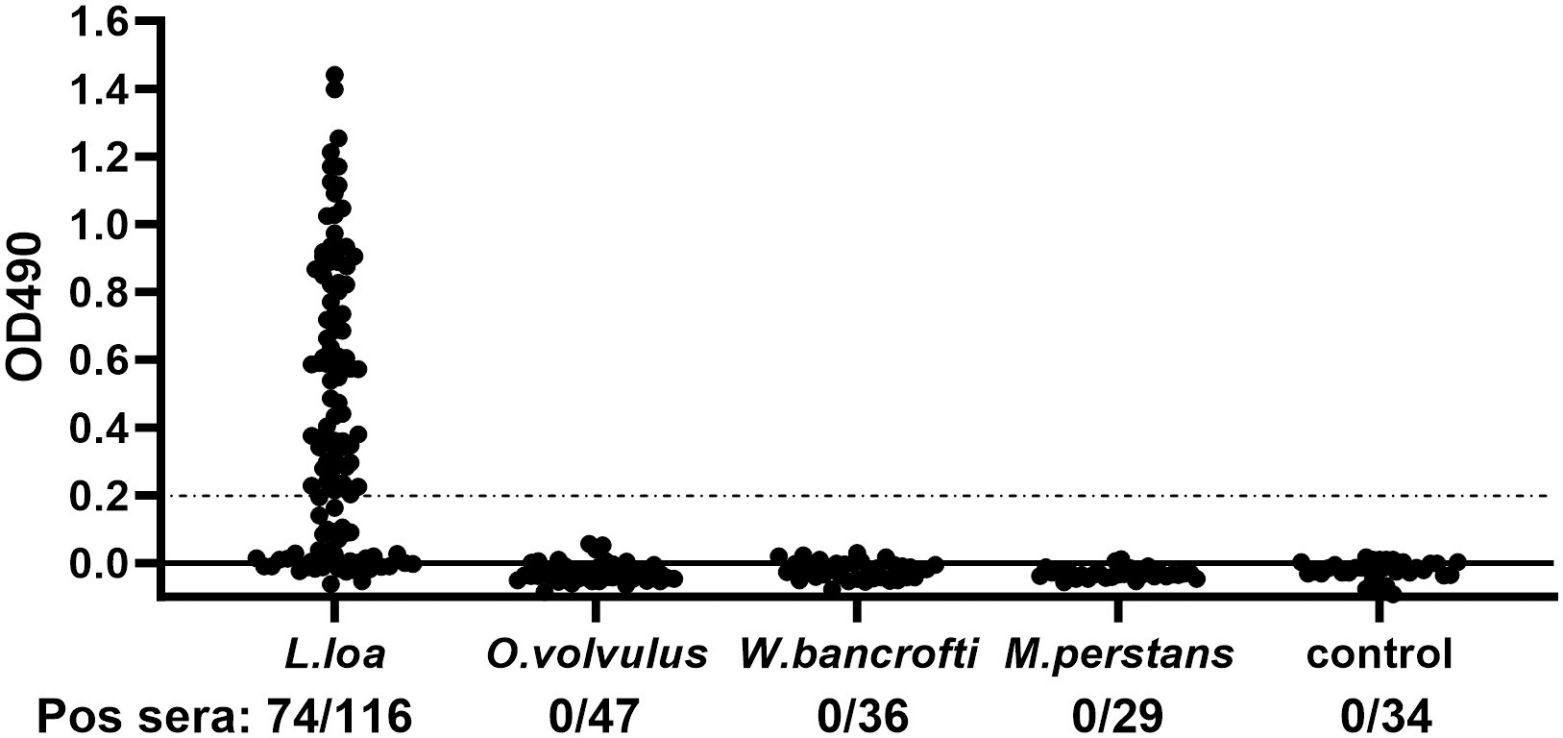
Sensitivity and Specificity of Ll-Bhp-1 antigen-capture ELISA. Graph shows the Ll-Bhp-1 antigen-capture ELISA OD_490_ values with sera from people with the indicated filarial infection or from non-endemic control sera. The OD value positivity cutoff of 0.2 is indicated by the dotted black line.

We used the optimized ELISA to test sera from 116 loiasis patients described in Table 1. We detected Ll-Bhp-1 in 74 of 116 (63.8%) samples (Fig 4). These people had daytime *L*. *loa* Mf counts that ranged from 140 to 159,800 Mf/ml based on thick blood smear. We also tested sera from people infected with other filarial parasites (Fig 4). None of the 47 sera from patients with *O*. *volvulus* infection had detectable Ll-Bhp-1. These sera were from patients with skin snip Mf counts between 2 and 1500 Mf per mg of skin. Similarly, none of 36 sera from patients with *W*. *bancrofti* infection had detectible Ll-Bhp-1. These sera were from patients with Mf counts that ranged from 2 to 5960 per ml by membrane filtration of 1 ml of blood. Some of the *W*. *bancrofti* samples from India were assessed by thick blood smear rather than filtration and those samples were from people with Mf counts of 7 to 142 per 20 μl thick smear. Thus, we tested samples with a range of Mf densities but saw no cross-reactivity with onchocerciasis or lymphatic filariasis samples in the Ll-Bhp-1 ELISA.

In our loiasis serum cohort, 19 of the 116 (16%) loiasis samples were from individuals co-infected with *Mansonella perstans*. The *O*. *volvulus* samples we utilized were from an area known to have *M*. *perstans* infection as well. None of the *O*. *volvulus* serum samples tested positive for Ll-Bhp-1, suggesting that the Ll-Bhp-1 ELISA does not detect antigen in samples from people with *M*. *perstans* infection. To confirm this, we tested sera from 29 patients who had documented *M*. *perstans* infection, and none had detectible Ll-Bhp-1 in our assay (Fig 4). We also tested non-endemic control sera from the USA and none of the 34 samples had detectible Ll-Bhp-1. Therefore, the Ll-Bhp-1 ELISA described here appears to be highly specific for loiasis.

**Fig 5:**
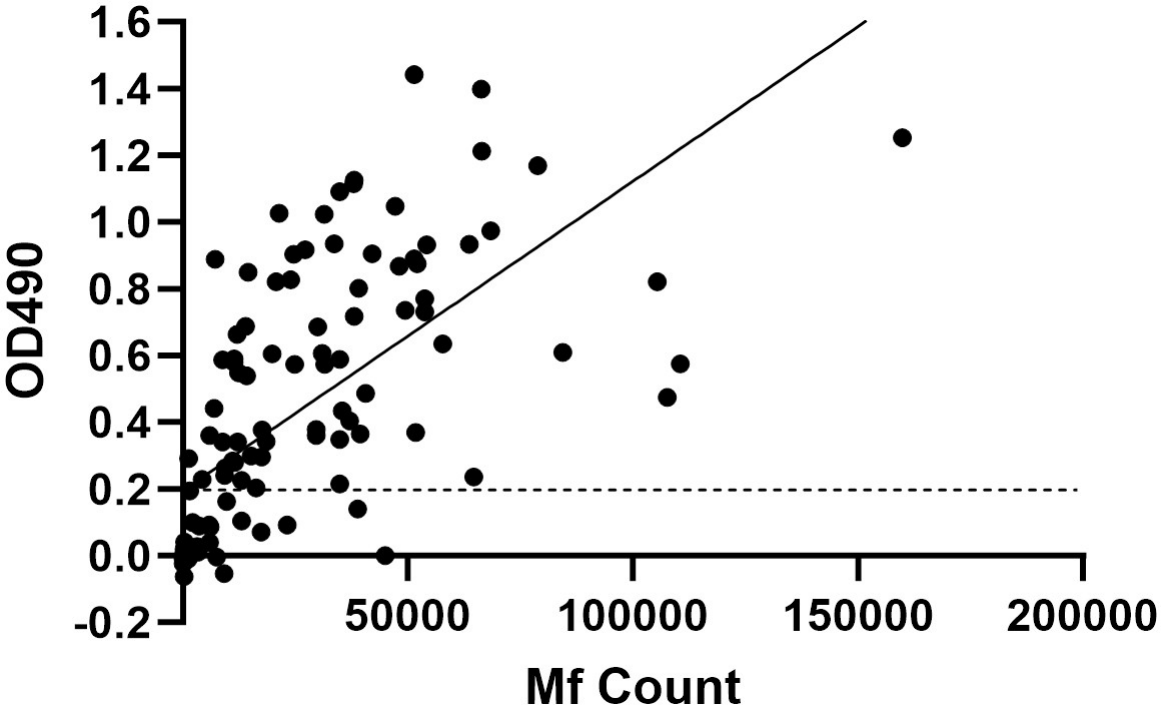
Ll-Bhp-1 antigen-capture ELISA results correlate with microfilarial density. Graph shows the Ll-Bhp-1 antigen-capture ELISA OD_490_ values plotted against the *L*. *loa* Mf count. The OD value positivity cutoff of 0.2 is indicated by the dotted black line. The best fit line is shown in black.

There was a large range in ELISA OD values from different participants. We tested whether Ll-Bhp-1 ELISA results correlate with *L*. *loa* microfilaremia and found the OD values were significantly correlated with *L*. *loa* Mf count (Spearman rank, r = 0.7981, p<0.0001) (Fig 5).

Given the strong correlation between Mf count and ELISA OD results, we were interested in how Mf count impacts the sensitivity of the Ll-Bhp-1 ELISA. This is especially important given that, for the “test-and-not-treat” approach to work, the test should accurately identify people with loiasis and a high Mf burden who are at increased risk of serious adverse events following ivermectin treatment, e.g., >20,000 Mf/ml. Indeed, the sensitivity of the assay was higher for samples with higher Mf counts (Table 2).

**Table 2:** Ll-BHp-1 antigen-capture ELISA results based on microfilarial density. This table demonstrates the percentage of *L*. *loa* serum samples that tested positive for Ll-Bhp-1 by ELISA, for people with different ranges of blood *L*. *loa* Mf counts.

	# positive/# tested	Sensitivity (%)
**All samples tested**	74/116	63.8
**Mf count <1,000 Mf/ml**	0/23	0
**Mf count 1,000–7,999 Mf/ml**	5/17	29.4
**Mf count 8,000–19,999 Mf/ml**	21/25	84
**Mf count 20,000–49,999 Mf/ml**	29/32	90.6
**Mf count >50,000 Mf/ml**	19/19	100

## Discussion

Diagnosis of loiasis is challenging for many reasons. In the past, surveys used the Rapid assessment procedure for loiasis (RAPLOA), a survey method that uses a history of eyeworm or Calabar swelling to identify loiasis patients [42,43]. However, many people with *L*. *loa* infection lack these classic clinical symptoms of loiasis. Antibody-based diagnostics are problematic in that they are most sensitive in those with few or no Mf [15]. Furthermore, available antibody tests for loiasis do not differentiate current from past infection [44]. Another diagnostic approach is based on detection of *L*. *loa* Mf in thick smears of blood collected during the day. This method requires equipment, electricity, and skilled technicians and microscopists, and it is difficult to implement on a large scale. The LoaScope is a mobile device that can quantify microfilarial load in blood and has the potential to ease some of the burden of Mf quantification [18]. However, LoaScopes have not been deployed on a country level scale to date. Mf detection by either LoaScope or microscopy fails to detect occult or amicrofilaremic loiasis infections, which are common [44]. Therefore, there is ongoing need for improved loiasis diagnostics, especially antigen tests. New point-of-care diagnostics could facilitate loiasis mapping and the “test and not treat” approach for LF and onchocerciasis elimination campaigns which use ivermectin. A point-of-care antigen detection assay could be a beneficial diagnostic tool to be used in conjunction with the LoaScope. It could be used for both loiasis mapping and to identify people with highly microfilaremic *L*. *loa* infection who are at increased risk of serious adverse events after ivermectin treatment.

The “test-and-not-treat” approach for MDA is an interesting strategy to prevent serious adverse reactions to ivermectin during LF and onchocerciasis elimination campaigns in Central Africa where loiasis is endemic. The prototype antigen-capture ELISA for native Ll-Bhp-1 antigen described here has excellent specificity and very high sensitivity in people with highly microfilaremic infections. A field-appropriate version of this assay could be useful for loiasis diagnosis and as a tool to exclude high risk people from mass treatment programs. Further work is needed to determine whether this test can be used to differentiate between people with light and heavy *L*. *loa* blood Mf counts, or if it can identify those with occult or amicrofilaremic loiasis.

Little is known about the biology of *B*. *malayi* BmR1, or its homologues in other filarial species. We have demonstrated that *L*. *loa* has two copies of each of the duplicated genes in the gene family that encodes this protein. The similarity of the Ll-Bhp-1 paralogues in *L*. *loa* is consistent with the hypothesis that these genes were created via gene duplication. Gene duplication is known to occur in filarial nematodes, and it has been noted that genes related to parasitism have often undergone gene duplication [45,46]. Furthermore, given the presence of 4 homologous genes, it is possible that *L*. *loa* parasites produce more of these proteins than homologues produced by other filarial worms. This factor, along with the high Mf counts that are common in loiasis, may explain the high sensitivity and specificity of this assay. Relatively low amino acid identity between Ll-Bhp-1 and the homologous proteins in other filarial species likely contributes to specificity as well.

Ll-Bhp-1 was predicted to be a microfilarial-associated protein, given the expression pattern of BmR1, its homologue in *B*. *malayi* [47,48]. Our immunohistochemistry results showed that this antigen was present in Mf, but also in various locations within adult female worms. The presence of Ll-Bhp-1 in secretory granules may explain the presence of this protein in extracellular vesicles from *L*. *loa* infected sera samples [20]. Extracellular vesicles are increasingly understood to be an important aspect of host-pathogen interactions and immune modulation [49,50]. It is possible these proteins may have interesting biologic functions. Further work is needed to investigate this family of proteins, their expression and their role in parasite physiology and host-parasite interactions.

High-level antigenemia, or antigen excess, may explain the low prevalence of antibodies to Ll-Bhp-1 in people with loiasis [21]. This would be consistent with our observation that the sensitivity of the Ll-Bhp-1 antigen-capture assay was improved by pretreating samples with EDTA and heat to release antigen from immune complexes and/or vesicles.

We are encouraged by the high sensitivity and specificity of this prototype ELISA based on polyclonal antibodies to recombinant Ll-Bhp-1. In subsequent research, we will test more samples to further validate the Ll-Bhp-1 antigen-capture ELISA. We will test samples from people with amicrofilaremic loiasis to assess the sensitivity of our assay in this population. We will also determine whether monoclonal antibodies can improve the sensitivity of this assay without sacrificing specificity. This has been seen with other filarial antigen diagnostics [35]. Further work is needed to optimize this assay for various use cases. For example, if we can use monoclonal antibodies to increase assay sensitivity to detect Ll-Bhp-1 in people with low *L*. *loa* Mf counts, this assay could be useful for diagnosis of loiasis in individuals. Such an assay could facilitate treatment to prevent adverse outcomes associated with longstanding untreated loiasis [1–6]. It could also be used for loiasis mapping efforts. On the other hand, an assay specific only for those with dangerously high *L*. *loa* Mf counts, e.g. > 20,000 Mf/ml, would be useful for mapping areas at high risk for serious adverse events following MDA of ivermectin. It would also be useful for the “test and not treat” approach to ivermectin MDA during LF and onchocerciasis elimination campaigns. We could potentially use the assay in conjunction with the LoaScope, to identify those with loiasis and decrease the number of people needed to be assessed by LoaScope. While the LoaScope depends on blood samples collected mid-day to assess the peak microfilaremia, antigen tests likely work independent of blood collection time and could be beneficial for testing during other times of day. Further work will be needed to see if we can transition this ELISA to a lateral flow-based assay platform, and if immune complexes would need to be dissociated for antigen detection in that format. We would also assess different sample preparation techniques that do not use heat to facilitate assay use in the field. In conclusion, we believe that assays for Ll-Bhp-1 have the potential to substantially improve loiasis diagnosis and efforts to eliminate important co-endemic filarial infections in Central Africa.

## Supporting information

S1 Fig Analysis of genomic structure for target proteins of interest with high sequence similarity. (**A**) A schematic summarizing the BLAST protein sequence similarity (%) and genomic positions for the four target genes (on *L*. *loa* genomic Scaffolds scf7180000007487_1 and scf7180000007489_1; "Scaffold 7487" and "Scaffold 7489", respectively). (**B**) Detailed genomic features including exons and introns for genes (annotated with "EN70" sequence IDs) and repeat sequences and regions of low complexity (annotated by repeat family IDs or no annotation for smaller regions). Mauve genomic alignments (red and green) indicate conserved genomic regions free from genome rearrangements ("Locally Collinear Blocks"; LCBs).
(TIF)

S2 Fig Integrative genomics viewer visualization. IGV visualization of long PacBio genomic read alignments to *L*. *loa* genomic regions of interest with similar gene sequences (on *L*. *loa* genomic Scaffolds scf7180000007487_1 and scf7180000007489_1; "Scaffold 7487" and "Scaffold 7489", respectively). Genomic scaffold positions, read depths, individual read positions and genomic features are shown.
(TIF)

S3 Fig Ll-Bhp-1 antigen-capture ELISA ROC. Graph shows the ROC curve for the Ll-Bhp-1 antigen-capture ELISA. The area under the curve is 0.9748 with p<0.0001.
(TIF)

S4 Fig Ll-Bhp-1 antigen-capture ELISA. Graphs show Ll-Bhp-1 ELISA data. The OD value positivity cutoff of ≥ 0.2 is indicated by the dotted black line. **A.** ELISA data for recombinant Ll-Bhp-1, either native or after EDTA/heat treatment. B. ELISA data for sera from loiasis patients or non-endemic controls with and without EDTA/heat or low pH glycine treatment.
(TIF)
