# Supplementary figures and images for: A novel antigen biomarker for detection of high-level of Loa loa microfilaremia
Source: PLoS Negl Trop Dis. 2024 Sep 3;18(9):e0012461. doi: 10.1371/journal.pntd.0012461 (PMC11398663; doi:10.1371/journal.pntd.0012461)

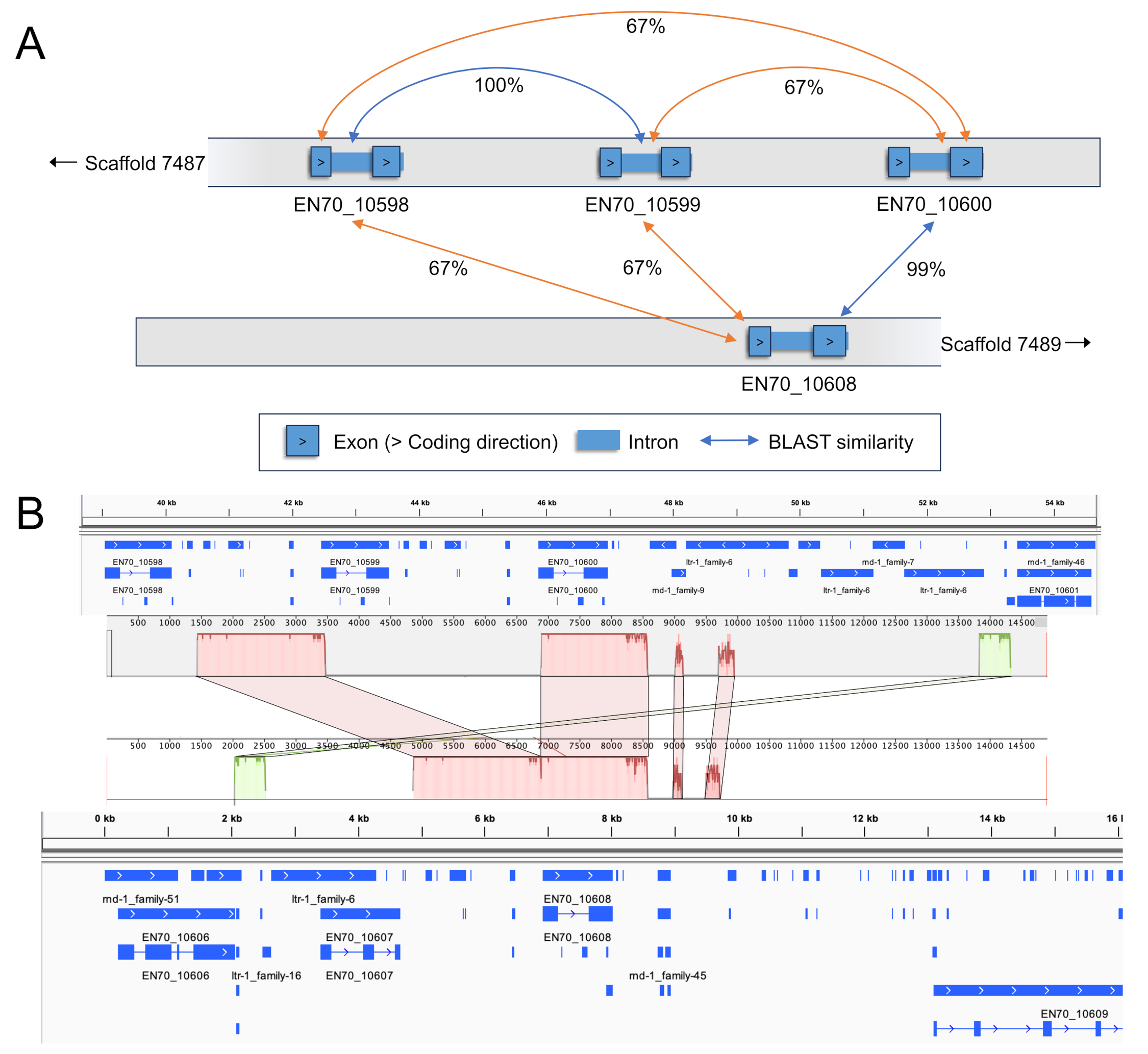

Supplement: S1 Fig — (A) A schematic summarizing the BLAST protein sequence similarity (%) and genomic positions for the four target genes (on L. loa genomic Scaffolds scf7180000007487_1 and scf7180000007489_1; "Scaffold 7487" and "Scaffold 7489", respectively). (B) Detailed genomic features including exons and introns for genes (annotated with "EN70" sequence IDs) and repeat sequences and regions of low complexity (annotated by repeat family IDs or no annotation for smaller regions). Mauve genomic alignments (red and green) indicate conserved genomic regions free from genome rearrangements ("Locally Collinear Blocks"; LCBs). (TIF) [file pntd.0012461.s001.tif]

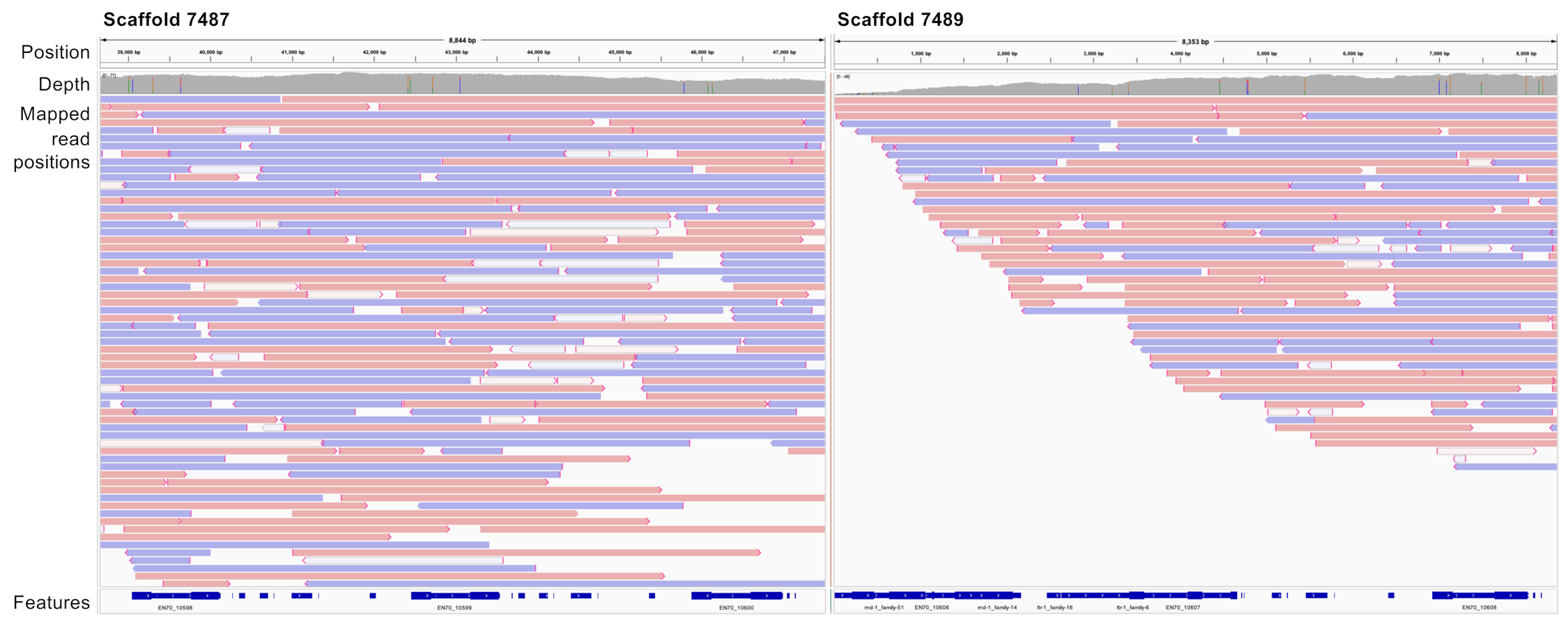

Supplement: S2 Fig — IGV visualization of long PacBio genomic read alignments to L. loa genomic regions of interest with similar gene sequences (on L. loa genomic Scaffolds scf7180000007487_1 and scf7180000007489_1; "Scaffold 7487" and "Scaffold 7489", respectively). Genomic scaffold positions, read depths, individual read positions and genomic features are shown. (TIF) [file pntd.0012461.s002.tif]

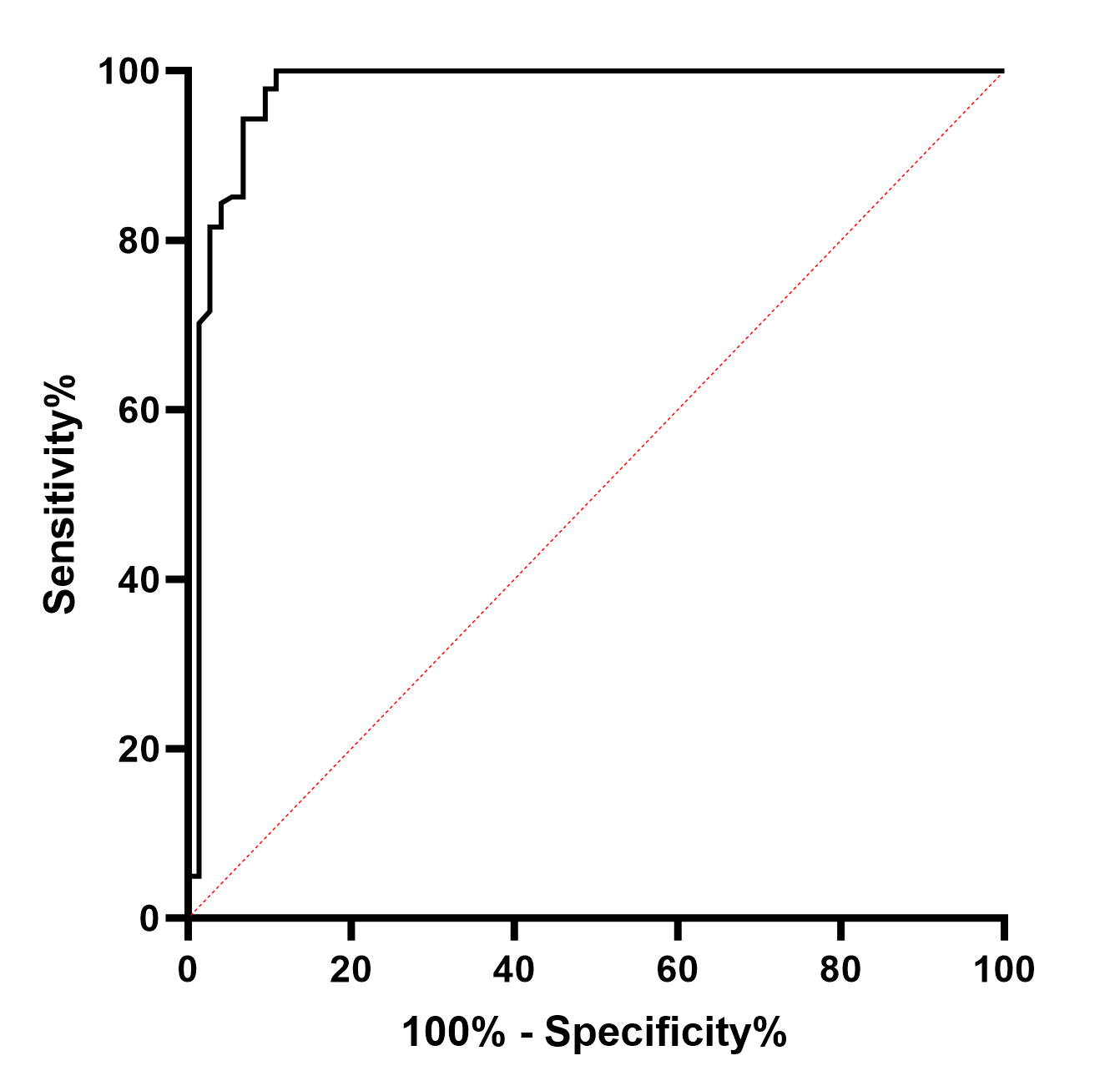

Supplement: S3 Fig — Graph shows the ROC curve for the Ll-Bhp-1 antigen-capture ELISA. The area under the curve is 0.9748 with p<0.0001. (TIF) [file pntd.0012461.s003.tif]

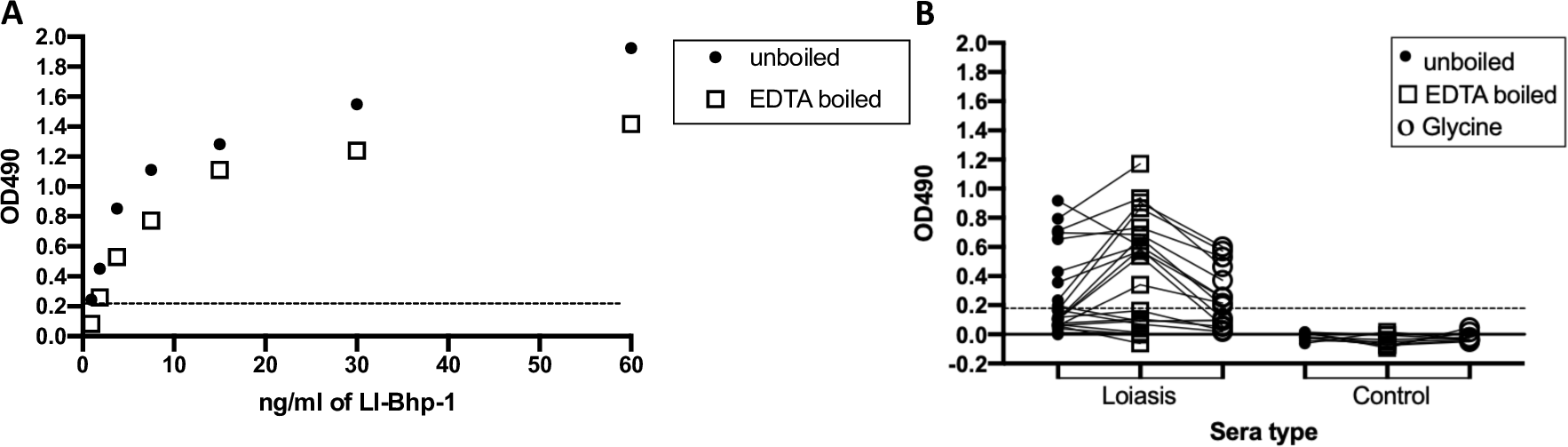

Supplement: S4 Fig — Graphs show Ll-Bhp-1 ELISA data. The OD value positivity cutoff of ≥ 0.2 is indicated by the dotted black line. A. ELISA data for recombinant Ll-Bhp-1, either native or after EDTA/heat treatment. B. ELISA data for sera from loiasis patients or non-endemic controls with and without EDTA/heat or low pH glycine treatment. (TIF) [file pntd.0012461.s004.tif]
